# Development and Validation of a Next-Generation Sequencing Assay for *BRCA1* and *BRCA2* Variants for the Clinical Laboratory

**DOI:** 10.1371/journal.pone.0136419

**Published:** 2015-08-21

**Authors:** Charles M. Strom, Steven Rivera, Christopher Elzinga, Taraneh Angeloni, Sun Hee Rosenthal, Dana Goos-Root, Martin Siaw, Jamie Platt, Cory Braastadt, Linda Cheng, David Ross, Weimin Sun

**Affiliations:** 1 Department of Genetics, Quest Diagnostics Nichols Institute, San Juan Capistrano, CA, United States of America; 2 Athena / Quest Diagnostics, Marlborough, MA, United States of America; 3 Celera / Quest Diagnostics, Alameda, CA, United States of America; Ohio State University Medical Center, UNITED STATES

## Abstract

The objective of this study was to design and validate a next-generation sequencing assay (NGS) to detect *BRCA1* and *BRCA2* mutations. We developed an assay using random shearing of genomic DNA followed by RNA bait tile hybridization and NGS sequencing on both the Illumina MiSeq and Ion Personal Gene Machine (PGM). We determined that the MiSeq Reporter software supplied with the instrument could not detect deletions greater than 9 base pairs. Therefore, we developed an alternative alignment and variant calling software, Quest Sequencing Analysis Pipeline (QSAP), that was capable of detecting large deletions and insertions. In validation studies, we used DNA from 27 stem cell lines, all with known deleterious *BRCA1* or *BRCA2* mutations, and DNA from 67 consented control individuals who had a total of 352 benign variants. Both the MiSeq/QSAP combination and PGM/Torrent Suite combination had 100% sensitivity for the 379 known variants in the validation series. However, the PGM/Torrent Suite combination had a lower intra- and inter-assay precision of 96.2% and 96.7%, respectively when compared to the MiSeq/QSAP combination of 100% and 99.4%, respectively. All PGM/Torrent Suite inconsistencies were false-positive variant assignments. We began commercial testing using both platforms and in the first 521 clinical samples MiSeq/QSAP had 100% sensitivity for *BRCA1/2* variants, including a 64-bp deletion and a 10-bp insertion not identified by PGM/Torrent Suite, which also suffered from a high false-positive rate. Neither the MiSeq nor PGM platform with their supplied alignment and variant calling software are appropriate for a clinical laboratory *BRCA* sequencing test. We have developed an NGS *BRCA1/2* sequencing assay, MiSeq/QSAP, with 100% analytic sensitivity and specificity in the validation set consisting of 379 variants. The MiSeq/QSAP combination has sufficient performance for use in a clinical laboratory.

## Introduction

Every year, more than 200,000 new cases of breast cancer are diagnosed in the United States [1]. Of these, approximately 2% to 5% are associated with loss-of-function variants in the *BRCA1* or *BRCA2* genes [1–4]. With the exception of Ashkenazi-Jewish women, who have a 2% to 5% carrier frequency for 3 founder mutations in *BRCA1* and *BRCA2* [5], the estimated carrier frequency in the general population is 1:300 for *BRCA1* [6] and 1:800 for *BRCA2* [5]. Patients with deleterious mutations in either the *BRCA1* or *BRCA2* gene have a 50% to 80% lifetime risk of developing breast cancer and a 20% to 40% lifetime risk of developing ovarian cancer [1–4,7–10]. Triple-negative breast cancers—those that do not express estrogen receptor, progesterone receptor, or Her2/neu and are characterized as being more aggressive—account for 15% to 20% of all breast cancers; they are associated with *BRCA* mutations at frequencies between 4% and 42%, depending on the characteristics of the study population (eg, proportion of women who are Ashkenazi Jewish) [11].

The National Comprehensive Cancer Network (NCCN) has developed guidelines for assisting healthcare providers in identifying patients and family members at high risk of breast and ovarian cancer and who may benefit from cancer genetic risk assessment [12]. Genetic risk assessment can include genetic testing but is a dynamic counseling process. Determining whether a woman with breast cancer is *BRCA1/2* positive can assist in appropriate counseling regarding increased surveillance and the risks and benefits of undergoing contralateral mastectomy and/or salpingo-oophorectomy, both of which have been shown to be protective against breast cancer [13]. Identifying a deleterious *BRCA1/2* variant in a patient can also be helpful to family members, who may need access to genetic counseling and testing to assess their cancer risk and identify appropriate management. The American Society of Breast Surgeons recommends *BRCA1/2* testing for individuals from high-risk populations, including those with early onset breast cancer (diagnosed before age 50); two primary breast cancers, either bilateral or ipsilateral; family history of early onset breast cancer; male breast cancer; personal or family history of ovarian cancer (particularly nonmucinous types); Ashkenazi (Eastern European) Jewish heritage in the setting of a newly diagnosed breast cancer or family history of breast cancer; previously identified *BRCA1* or *BRCA2* mutation in the family; triple-negative breast cancer at ≤60 years of age; or pancreatic cancer associated with a family history of hereditary breast and ovarian related cancer [14].

Comprehensive *BRCA* testing consists of sequencing all the coding exons and the splice junction regions of *BRCA1* and *BRCA2*, plus analysis of large rearrangements [12]. In our laboratory we are performing the large rearrangement analysis using Multiplex Ligation Probe Amplification (MLPA) kits purchased from MRC Holland. This article describes only the sequencing based part of our comprehensive BRCA test. PCR-based sequencing methods, including Sanger sequencing and next-generation sequencing (NGS) systems that use PCR amplification, may yield false-negative results due to allele drop-out when polymorphisms are present in amplification or sequencing primer sequences [14]. The use of bait tile library exon capture followed by NGS can avoid this potential cause of false-negative testing. Bait tiles are biotinylated 125-bp RNA molecules used to capture relevant fragments. Since the bait tiles are 100 bases longer than typical PCR or sequencing primers and RNA/DNA hybrids are stronger than DNA/DNA hybrids, polymorphisms are less likely to interfere with the exon capture. A second major advantage of bait tile capture versus PCR based sequencing methods is the avoidance of false positive results due to clonal bias in PCR or library formation. We developed an NGS-based assay using bait tile exon capture for detection of *BRCA1/2* variants in a reference laboratory. Two different NGS platforms were employed: the Illumina MiSeq System and the Life Technologies Ion Torrent Personal Genome Machine. Here we report the validation of this assay, results from the first 521 clinical samples obtained using both NGS platforms, and an additional 1006 results obtained using duplicate MiSeq runs. We have not previously offered BRCA testing in our laboratory. Our complete offering includes large rearrangement testing for all coding expos using MLPA.

## Materials and Methods

**Fig 1:**
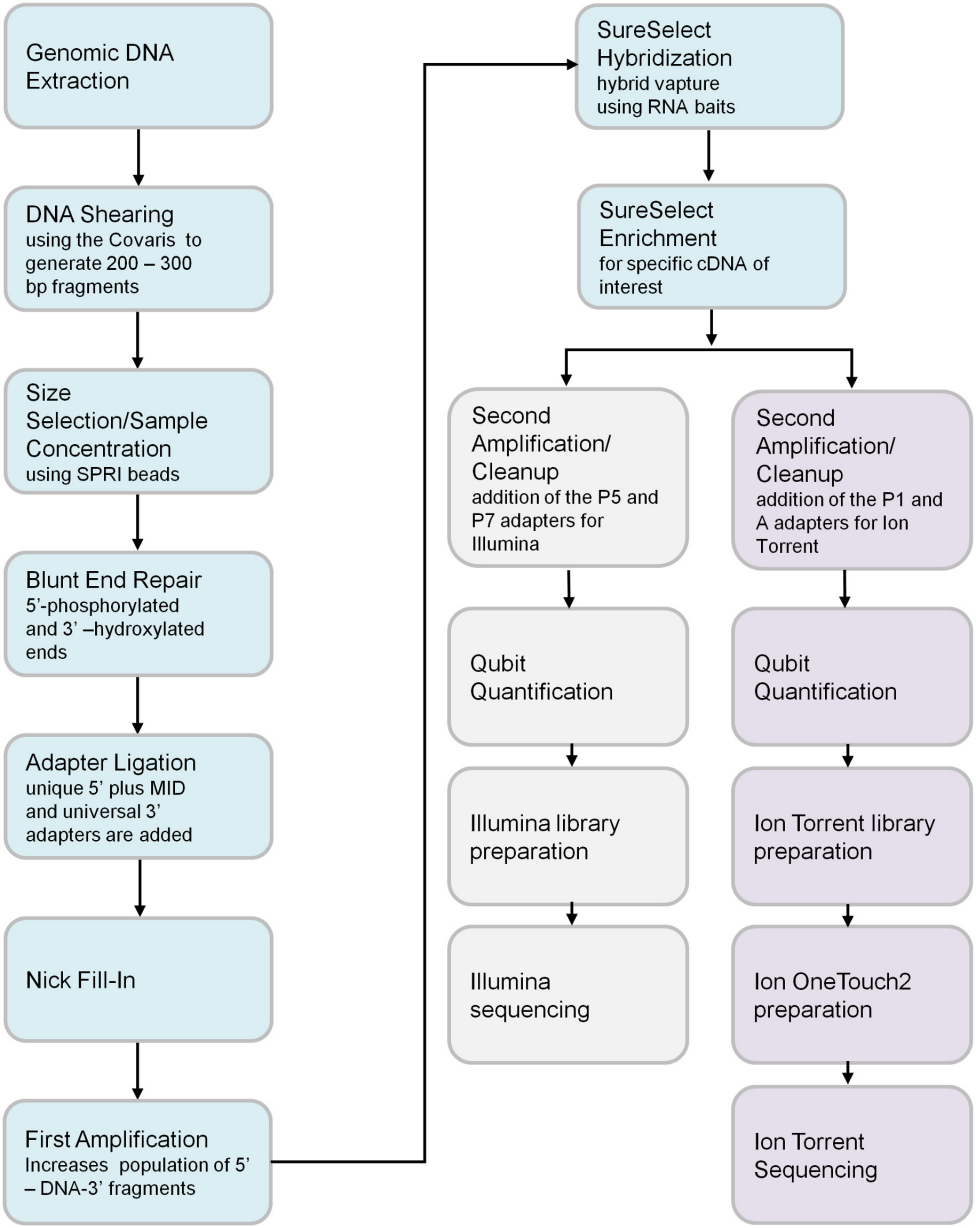
Schematic of the work flow for the next-generation sequencing (NGS) *BRCA1/ BRCA2* assay using both the MiSeq and the Personal Gene Machine (PGM) platforms. For the MiSeq platform, variant calling was performed initially with the vendor-supplied MiSeq Reporter software and then with the Quest Sequencing Analysis Pipeline (QSAP). For the PGM platform, the vendor-supplied Torrent Suite variant-calling software was used.

Fig 1 illustrates the general overview of the NGS assay for detection of *BRCA1* and *BRCA2* variants.

### DNA Samples

DNA samples from cell lines with known deleterious variants in *BRCA1* (n = 21; Table 1) or *BRCA2* (n = 6; Table 1) were purchased from the Coriell Mutant Cell Repository (Camden, NJ). These reference samples contained both pathogenic and nonpathogenic variants. We also obtained blood samples from 67 unaffected individuals previously untested for *BRCA* mutations and performed Sanger sequencing to determine the presence of *BRCA1* or *BRCA2* sequence variations. In all, 352 benign variants were identified in the volunteer population and were used in the technical validation. All 67 subjects provided written informed consent. The protocol for this study was approved by the Western Institutional Review Board.

**Table 1:** *BRCA1* and *BRCA2* Variants in the 27 Coriell Cell Line Reference Samples.

	dbSNP HGVS names	
Sample	NM_007300.3	NP_009231.2
***BRCA1***		
GM13711	c.3119G>A	p.Ser1040Asn
GM13715	c.5326_5327insC	p.Ser1776delinsSerProfs
GM14634	c.4065_4068delTCAA	p.Asn1355_Gln1356delinsLysfs
GM14636	c.5621_5622insA	p.Tyr1874delinsTerProfs
GM14637	c.4327C>T	p.Arg1443Ter
GM14638	c.213-11T>G	-
GM14684	c.797_798delTT	p.Val266 = fs
GM14090	c.66_67delAG	p.Leu22_Glu23delinsLeuValfs
GM14092	c.5201T>C	p.Val1734Ala
GM14093	c.1204delG	p.Glu402Serfs
GM14094	c.1175_1214del40	p.Leu392_Ser405delinsGlnfs
GM14095	c.5200delG	p.Val1734Terfs
GM14096	c.3481_3491delGAAGATACTAG	p.Glu1161_Ser1164delinsPhefs
GM14097	c.181T>G	p.Cys61Gly
GM13714	c.5382_5383insC	p.Asn1795Glnfs
GM13713	c.3748G>T	p.Glu1250Ter
GM13712	c.2155_2156insA	p.Lys719delinsLysArgfs
GM13710	c.4327C>G	p.Arg1443Gly
GM13709	c.2068delA	p.Lys690 = fs
GM13708	c.4752C>G	p.Tyr1584Ter
GM13705	c.3756_3759delGTCT	p.Leu1252_Ser1253delinsLeufs
***BRCA2***		
GM14170	c.5946delT	p.Ser1982Argfs
GM14622	c.6275_6276delTT	p.Leu2092Profs
GM14623	c.125A>G	p.Tyr42Cys
GM14624	c.5718_5719delCT	p.Asn1906_Ser1907 = fs
GM14626	c.9976A>T	p.Lys3326Ter
GM14639	c.6198_6199delTT	p.Val2066_Ser2067delinsValHisfs

All mutations were detected by NGS with the PGM system and the MiSeq system (with QSAP variant calling) software, as well as by Sanger sequencing.

#### DNA Preparation

Genomic DNA from peripheral blood cells was isolated in 96-well microtiter plates using a Roche Magnapure system from Roche Molecular Systems (Indianapolis, IN) per the manufacturer’s instructions. Genomic DNA was randomly sheared to an average size of 250 base pairs using adaptive focused acoustics technology (E220 Focused Ultra-Sonicator, Covaris Inc., Woburn, MA) according to the manufacturer’s instructions.

#### Concentration via SPRI Beads and PEG/Sodium Chloride Mix

Immediately after shearing, the DNA was concentrated 2-fold and DNA molecules with inadequate sizes were removed. This was accomplished with SPRI (solid-phase reversible immobilization) beads (AMPure Beads, Agencourt, Beverley, MA). The beads were suspended in a solution of polyethylene glycol (PEG), EDTA.

#### DNA End Repair

The ends of the DNA molecules were repaired prior to adaptor ligation. This was accomplished using a DNA polymerase that has both 5’ to 3’ polymerase activity and 3’ to 5’ exonuclease activity, thereby filling in 5’ overhangs and removing 3’ overhangs to generate blunt ends. In addition, the 5’ end of the DNA fragments were also phosphorylated in this process.

#### Adaptor Ligation and Nick Repair

Each 5’ adaptor contains a unique molecular identification (MID) sequence (barcode) that is used to identify individual DNA samples. In addition, it contains a portion of the P5 sequence. The 3’ adaptor is universal to all specimens and contains a portion of the P7 MiSeq sequence. Neither adaptor is 5’ phosphorylated. A short complimentary oligonucleotide for each of the adaptors is also included in the ligation reaction to ensure that the adaptors are only ligated to the DNA fragments and not to themselves. During ligation the molar ratios of the two adaptors are equal to each other but are in excess in comparison to the fragmented DNA. Following this procedure, approximately half of all ligation products are the preferred species, namely: 5’-(P5)-MID-*BRCA*_GeneDNA-universal (P7)-3’. The samples are cleaned using SPRI beads as described above, and the nicks at the ligation site are repaired by a DNA polymerase. The polymerase adds nucleotides at the nick site creating a primer binding site for PCR amplification.

#### Pre-hybridization Amplification

In order to increase the ratio of adapted DNA fragments non-allele specific PCR was performed. The primers used were complementary to the 5’ and the 3’ adaptor sequences.

#### Target Enrichment Through Exon Capture

All bar coded patient DNA fragments were pooled to create a “library,” and were added to a hybridization reaction mixture and incubated for 12 hours at 65°C. This mixture contained the biotinylated RNA baits. The baits were complimentary to *BRCA1* and *BRCA2* genes (exonic regions and splice junction sites, and a selected intronic region) to allow hybridization to the appropriate patient DNA fragments. After the hybridization, the library was combined with streptavidin coated beats to adsorb the biotinylated RNA baits. The library-RNA bait hybrids were washed at 70°C to remove the non-*BRCA* DNA.

#### Second Nonspecific Amplification

Additional sequences required for either the Illumina MiSeq (Illumina, San Diego, CA) or Ion Torrent Personal Gene Machine (PGM) (Life Technologies, Grand Island, NY) sequencing platforms were added to the 5’ and 3‘ adaptors using fusion primers. The DNA library was divided into two halves. One half was amplified with fusion primers (P5 and P7 sequences) that have a portion complementary to the 5’ and 3’ adaptors and add additional sequences for MiSeq sequencing and the other half was amplified with a set of primers (P1 and A sequences) that add additional sequences for PGM sequencing.

### Quantification by Qubit

The high sensitivity Qubit kit (Life Technologies), which uses an intercalating dye based method, was used to quantify DNA.

### Sequencing

The library was diluted so that amplification generated well-separated clusters of identical products from a single DNA molecule (clonal amplification) The MiSeq and PGM NGS protocols were performed according to the manufacturer’s protocols.

#### MiSeq

The single-stranded library was loaded into well 21 of the MiSeq sequencing cartridge. The instrument flushed the library through the flow cell where it hybridized to the antisense P5 and P7 oligonucleotides that are complimentary to the adaptors on the library. The library was diluted so that amplification generated well-separated clusters of identical products from a single DNA molecule (clonal amplification). This was accomplished by isothermal bridge amplification. Fluorophore-labeled nucleotide triphosphates were applied to the flow cell and then excited by a laser. The emission spectra was recorded by the MiSeq, and then the nucleotide blocker, which inhibited further synthesis, was cleaved, allowing for addition of the next nucleotide triphosphate. In this manner, fragments were sequenced.

#### PGM

The PGM uses emulsion PCR, the amplification inside of tiny water droplets floating in oil. Emulsion PCR is performed to get many copies of a single DNA molecule onto a single sequencing “bead” (clonal amplification). These beads are then used to generate the sequence. The beads used in the amplification solution are covered with covalently bound oligonucleotides that are antisense to the P1 sequence of the library. Micro-chambers are created by the Ion Torrent One Touch Instrument 2 (OT2, Life Technologies, Grand Island, NY), which carries out clonal amplification.

The strand of DNA that results from extension of the anti-sense P1 oligonucleotide is then hybridized at its 3’ end with a sequencing primer that binds at the anti-sense A oligonucleotide. DNA polymerase is added to the beads and then the beads are deposited into tiny pores on the surface of a computer chip-like surface. Each of the four dNTPs is then sequentially flowed in excess over the surface of the chip. The DNA polymerase extends the growing strand when the required nucleotide is made available. Whenever a nucleotide is added, a hydrogen molecule is released resulting in a pH change in the pore containing the sequencing bead. The magnitude of the pH change is approximately equal to the number of nucleotides incorporated and is detected and measured along with which of the four nucleotides that flowed through.

### Bioinformatics Processing

Following the sequencing reaction, sequence alignment and allele assignment was performed. Initially, for the MiSeq we used the MiSeq Reporter software supplied with the instrument. However, when it became clear that this combination consistently did not identify deletions larger than 9 bp, we developed our own, proprietary bioinformatics pipeline called QSAP. The *BRCA1/2* advanced sequencing bioinformatics modular workflow manages the sequence information from Illumina MiSeq FASTQ files to final reporting in the CLIA and CAP certified laboratory. The workflow uses the IDBS Biomolecular Hub (ID Business Solutions Ltd, Guildford, Surrey, UK) as well as customized visualization to manually review the results for nomination to the clinical report. The QSAP is the specialized portion of the overall workflow that integrates open source, in-house developed and licensed modules for sequence analysis. The analysis pipeline, using a high performance computing infrastructure, includes a Piccard tools, the Burrows Wheeler Aligner (BWA) for mapping and alignment to the hg19 genome, the Genome Analysis Tool Kit (GATK) (for de-duplication, Smith-Waterman realignment and variation calls), as well as a QC Metrics report generator (e.g. number of reads, mean coverage, minimum coverage) followed by result parsing and cataloging in purpose-built databases. The pipeline is designed to maximize the accuracy of variant calls, reduce time of analysis and permit ready access to sample Binary alignment/Map format (BAM) and Variant Call Format (VCF) files.

The de-identified VCF files were transferred to Ingenuity (Qiagen, Germantown, MD) for an automated preliminary assessment, and an annotated XML file was returned. The initial automated assessment leverages variant molecular classification (e.g. synonymous, missense, nonsense, frameshift), comprehensive clinical evidence (largely curated by scientists from peer-reviewed literature) and provides evidence-based clinical decision support that assists the initial classification of the observed variants. The preliminary automated assessment improves the turnaround time and maximizes the information used for the final clinical assessment of variants. The automated assessment has two additional fundamental features. First, the assessment was configured using Quest Diagnostics pre-defined scoring and classification rules with American College of Medical Genetics recommended guidelines being the central advisement combined with a compilation of evidence including the most recent literature. Second, the software provides reviewers with direct access to the relevant literature for variants as well as providing transparency to how the automated assessments were derived to facilitate review of primary data. Subsequent manual review through Ingenuity’s VCS web interface, additional locus-specific databases and confirmatory queries were carried out to complete the final classification.

For the PGM data, bioinformatics analyses were performed using the Torrent Suite software supplied with the instrument.

### Variant Assessment

Variant assessment is performed manually by a team of variant scientists (VS) according to the guidelines of the American College of Medical Genetics. VCF files are analyzed by the software program Alamut that provides genomic coordinents and SIFT *in vitro* functional analysis. The VS rechecks the quality metrics of the individual run and, if the run passes QC proceeds to the assessment. At this point the deletion / duplication results from the MLPA reactions are also reviewed. The VS then double checks the called variants to assure concordance with the IGV data. If variant identification is accurate, the variants are loaded from Alamut HT into a proprietary database called QuestIQ. The following fields are automatically filled by the Alamut HT software interface: Gene|Variant, Variant ID, Ref Seq, DNA level, Mutation type, Code Interpretation, PUC, Gene Code, Exon, Nucleotide, Change, Codon, Amino Acid, dbSNP rs#, dbSNP link, SIFT, Species conservation, Link to VUS analysis text, link to Splicing Report, MolGen accessions. The VS will then search for further information using the gene specific databases UMD, BIC, LOVD, IARC, ClinVar, ARUP, kConFab, HGMD, InSIGHT. This is followed by assessing the variant frequency using ESP and dbSNP. If applicable, post translation predictive databases with be used such as NetPhosk, NetPhos, ScanSite: S, T,Y phosphorylation predictions, Yin o Yang: O-linked GlcNac. Spicing predictions are made using linked software in Alamut HC, using the RefSeq database. The functional predictive programs SIFT and PolyPhen2 are then used.

Subsequently, a manual literature search is performed to determine if there is further supporting data on the particular variant using a Google search through Alamut, PubMed, ScienceDirect, and BioMed Central. All relevant results are entered into the IQDB database. The final variant classification is made according to the ACMG guidelines and the result entered into the IQDB database. Classifications are scored as Benign, Likely Benign,VUS, Likely Pathogenic, Pathogenic. This result is then passed to Director for Secondary review and report writing.

## Results

### Assay Development

**Fig 2:**
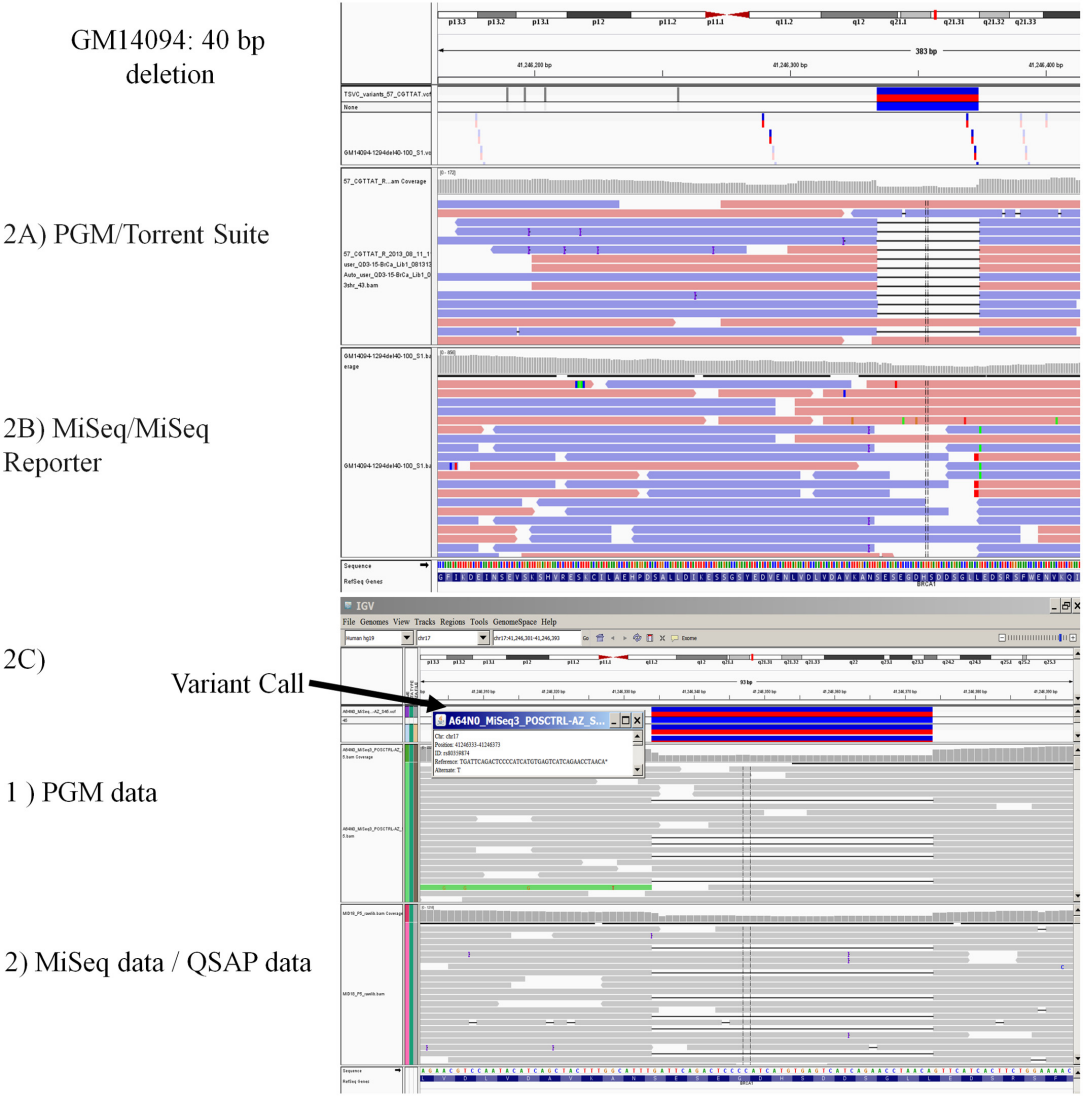
Alignment of a 40-bp deletion in *BRCA1* (deletion c.1175_1214del40) in a validation sample. The Integrative Genomics Viewer (IGV) graphic report shows detection of the mutation with the PGM platform with Torrent Suite variant calling (panel A) but not the MiSeq platform with MiSeq Reporter (panel B). Use of QSAP with the MiSeq platform allowed detection of the deletion (panel C).

During assay development, the MiSeq sequencing system using the supplied MiSeq Reporter software was unable to identify 2 of the pathological *BRCA1* variants in tested Coriell samples. Both variants were deletions of >9 bp: the 40-bp deletion c.1175_1214del40 and the 10-bp deletion c.3481_3491del10. These were the only deletions of >9 bp in these samples. We therefore developed a proprietary bioinformatics pipeline for alignment and allele assignment (QSAP) as described above. Fig 2 shows the alignment representation for the sample containing a 40-bp deletion. This deleterious mutation was identified by the PGM/Torrent Suite software but not the MiSeq/MiSeq Reporter software. However, the deletion was clearly identified when using MiSeq with QSAP software (Fig 2). Similar findings were seen for the 10-bp deletion, with MiSeq/MiSeq reporter consistently missing the deletion and the PGM/Torrent Suite and MiSeq/QSAP always identifying the deletion (data not shown). The MiSeq/QSAP and the PGM/Torrent Suite combinations both showed 100% sensitivity for the *BRCA1* and *BRCA2* variants in the validation set. We therefore entered technical validation using both platforms.

Since NGS sequencing errors can result from PCR or clonal amplification errors, we developed a QC metric to overcome these potential problems, taking advantage of the fact that random shearing leads to library clones that have different starting and ending positions. Therefore the bioinformatics analyses were able to differentiate among reads from the same versus different library clones. This enabled us to develop a minimum QC metric, whereby each targeted base must have high quality sequence from a minimum of 20 unique clones. Typically however, we were able achieve an “average depth of read” of 335 unique reads.

### Technical Validation: Intra-assay Precision

Intra-assay precision was established by analyzing the DNA extracted from 3 blood samples, in 5 replicates on each platform. Each sample had at least one *BRCA1* or *BRCA2* variant. All variants identified in each sample were 100% concordant within the 5 replicates when detected on the MiSeq/QSAP combination. However, the PGM/Torrent Suite platform exhibited a low level of random sequencing errors. Overall intra-assay concordance on the PGM instrument was only 96.2%. In 1 sample, a single base insertion was detected in the fifth replicate that was not detected in the other replicates. In addition, a benign variant was not identified in the fourth replicate. In another sample, the fifth replicate contained 4 SNPs that were not present in any of the other replicates, and one SNP was called in replicate 3 that was not called elsewhere.

In comparing the MiSeq/QSAP allele calls to the PGM/Torrent Suite calls, there were several discordances. One sample showed discordance at 2 SNP sites between the 2 platforms: PGM/Torrent Suite called an A insertion at position 32906554 on chromosome 13 in one of the replicates that was not identified in the remainder of the PGM/Torrent Suite replicates or on the MiSeq/QSAP platform. The PGM/Torrent Suite also did not detect rs1799949 at position 41245466 on chromosome 17 in one replicate. As a result, intra-specimen concordance was 88% between the 2 platforms for this specimen. The second sample was concordant on all variant calls from both platforms, resulting in a concordance of 100% between platforms. The third sample was discordant at 5 of 11 SNPs due to sequencing errors on PGM/Torrent Suite (1 of 5 replicates for each SNP), for a concordance rate of only 64%.

### Technical Validation: Inter-assay Precision

DNA from remnant laboratory samples from 67 presumably unaffected individuals, plus the 27 DNA specimens from Coriell, were analyzed in 3 replication set-ups. Two negative controls (a quality control blank [QCB]) and a no-template control [NTC]) were also included in each run. Libraries prepared for each run were detected on both the MiSeq/QSAP and PGM/Torrent Suite platforms. All variants detected in 3 replication runs from both the PGM and MiSeq instruments were verified by manual review using IGV (version 2.3.14). Specimens failing in 2 or more of the replication runs were excluded from the inter-assay variability assessment.

Fewer verified variants were detected on the PGM/Torrent Suite platform than on the MiSeq/QSAP platform, owing to higher assay failure rate on the former (Table 2). The inter-assay precision was 96.7% for the PGM/Torrent Suite and 99.4% for the MiSeq/QSAP (Table 2). Of note, discrepant calls detected among the 3 replicates on the MiSeq/QSAP platform represented false-positive results; most came from a single sample on a single replicate, likely indicating a problem with sample preparation or well contamination.

**Table 2:** Inter-Assay Concordance of Variant Calls vs. Sanger.

	PGM/Torrent Suite	MiSeq/QSAP
Concordant Calls	1550	2188
Discrepant Calls	53	13
Total Calls	1603	2201
% Concordance	96.7%	99.4%

Samples analyzed included 27 control DNA specimens with known deleterious mutations and 67 specimens from volunteers with a total of 352 benign variants.

### Failed Specimens

Specimen failure was defined as failure to achieve an average coverage depth of >40x at any exon. For the MiSeq instrument, there were no failures in replications 1 and 2 and 8 specimen failures in replication 3. Therefore, the overall failure rate was 8.5% (8/94) for replication 3, or 2.8% (8/282) overall. For the PGM, the failure rate was 9.6% (9/94) for replication 1, 13.8% (13/94) for replication 2, and 26.6% (25/94) for replication 3; the overall failure rate was 16.7% (47/282). All the failed specimens with low coverage were among the control specimens from consented subjects, possibly reflecting higher DNA quality in the Coriell DNA samples.

Four specimens failed in replication 3 on the MiSeq/QSAP platform and in all 3 replication runs on the PGM/Torrent Suite platform. This finding suggests specimen quality issues, although the same specimens were successfully sequenced for all regions in replications 1 and 2 on the MiSeq/QSAP platform. All the specimens that failed on the MiSeq/QSAP platform were from replication 3. The failure rate on the PGM/Torrent Suite platform was also highest for replication 3. This points to a sample preparation issue for that replication, as the sample libraries for both platforms were prepared together up to and through the hybrid capture step.

### Inter-platform Concordance

As there were more failures in the PGM runs than in the MiSeq runs, we were only able to verify a subset of discrepant variant calls from the MiSeq/QSAP platform with calls from the PGM/Torrent Suite platform. All 8 discrepant variant calls from MiSeq/QSAP were from replication 2. Four of the 8 were similarly observed in replication 2 on the PGM/Torrent Suite platform. In addition, 5 of the 8 variant calls were observed in a single sample.

### Analytic Sensitivity: Detection Limits

#### Limit of Blank (LOB)

The NTC and the QCB bar coded specimens were carried throughout the assay and handled identically to all other specimens. The number of reads mapped to the hg19 genome sequence was compared to the average aligned reads per sample. For the MiSeq/QSAP platform in replication 1, no reads were mapped to the human genome for either the NTC or the QCB. In replication 2, the QC blank had no reads but the NTC had 1,486 reads. This totaled 1.1% of the average number of reads on the plate and was well below the 20% threshold for an allele call. In replication 3, the NTC had 46 reads, representing 0.044% of the average number of reads in plate 1.

For the PGM/Torrent Suite platform, the NTC and QCB demonstrated 0.255% and 0.029% aligned reads in plate 1. In plate 2, the NTC had 9.2% of the average aligned reads while the QC blank had zero. The values for the 3^rd^ replication plate were 0.268% and 0.063% for the NTC and QCB, respectively. The NTC and QCB demonstrated an acceptably low overall number of aligned reads on both platforms. The aligned reads were either not detectable or well below our cutoff threshold of 20% for variant calls.

#### Limit of Detection (LOD)

We defined the LOD as the lowest DNA concentration (ng/μL) at which the average read depth over the exonic region was maintained at ≥40 reads per base. To determine the LOD, we undertook the following experiments. Two Coriell DNA samples, GM14094 and GM14096, and a single random DNA sample chosen from the 67 control individuals lacking pathogenic *BRCA* were serially diluted. On the MiSeq/QSAP platform, the control DNAs failed to achieve the required average read/coverage depth at 5 ng/μL demonstrating that the minimal sample input (LOD) for the MiSeq/QSAP platform must be greater than 5 ng/μL (all shearing reactions were carried out in 80 μL volumes). In addition, all variants were consistently called (i.e., 100% concordant) at each concentration for the 3 specimens above this lower limit. On the PGM/Torrent Suite platform, the samples failed at 5 ng/μL, demonstrating that the minimal sample input must also be greater than 5 ng/μL (400 ng of DNA). On both platforms, the 40-bp and 11-bp deletion mutations were successfully detected at all concentrations. However, only 99.96% of the called variants were concordant for the non-Coriell control sample. At 15 ng/μL, an insertion was called in the control DNA using the PGM/Torrent Suite platform that was not present in any of the other concentrations and was likely due to a sequencing error.

#### Accuracy

The 27 DNA specimens obtained from Coriell were included in this validation study, in 3 separate runs, on both MiSeq/QSAP and PGM/Torrent Suite platforms. All previously known *BRCA1* and *BRCA2* variants in the specimens were successfully detected by both platforms and in all 3 validation runs (i.e., 100% accuracy for cancer-associated mutations). In addition, we determined the overall accuracy of variant calls for the 352 benign sequence changes detected in the 67 control samples. There was only one missed call on the MiSeq/QSAP platform, which was due to low read depth (coverage). This error could have been avoided by adjusting the minimum depth requirement in our QC metric as this was implemented prior to going live with the assay. The PGM/Torrent Suite platform yielded 2 false-positive calls, one sequencing error, and 37 missed variant calls, most of which were observed in only one of the three validation runs. However, there were 4 variants not called by Ion Reporter, which were detected by manual review of the alignment software. Overall, the error rate was <0.1% (1/1056) for the MiSeq/QSAP platform and 3.7% (39/1056) for the PGM/Torrent Suite platform. With the adjusted QC parameters, the MiSeq/QSAP combination had 100% sensitivity and nearly 100% specificity. With manual review of all positive samples, the MiSeq/QSAP combination also achieved 100% specificity.

#### The First 521 Clinical Samples

For the initial clinical test release, mutation analyses were performed using both the MiSeq/QSAP platform and the PGM/Torrent Suite platform variant calling software. For samples with discrepant results on the 2 platforms, we either manually reviewed the cases to determine the cause of the discrepancy or retested the samples for confirmation. There were 35 discrepancies in the first 521 reported cases, with 34 due to PGM/Torrent Suite errors. The single MiSeq/QSAP platform sequencing error was a false-negative result for a benign polymorphism. Manual review of the alignment revealed that this was due to a combination of strand bias (19% variant) and low coverage. We then adjusted our QC parameters to take advantage of the fact that random shearing allows the filtering of duplicate reads. The QC acceptance metric requires that each base in each assay be analyzed from at least 20 independent reads. This typically resulted in an average depth ranging from several hundred to a few thousand. Using adjusted QC parameters, the MiSeq/QSAP combination had 100% sensitivity and 100% specificity. For all positive cases, the alignments are manually reviewed as a further quality measure.

**Fig 3:**
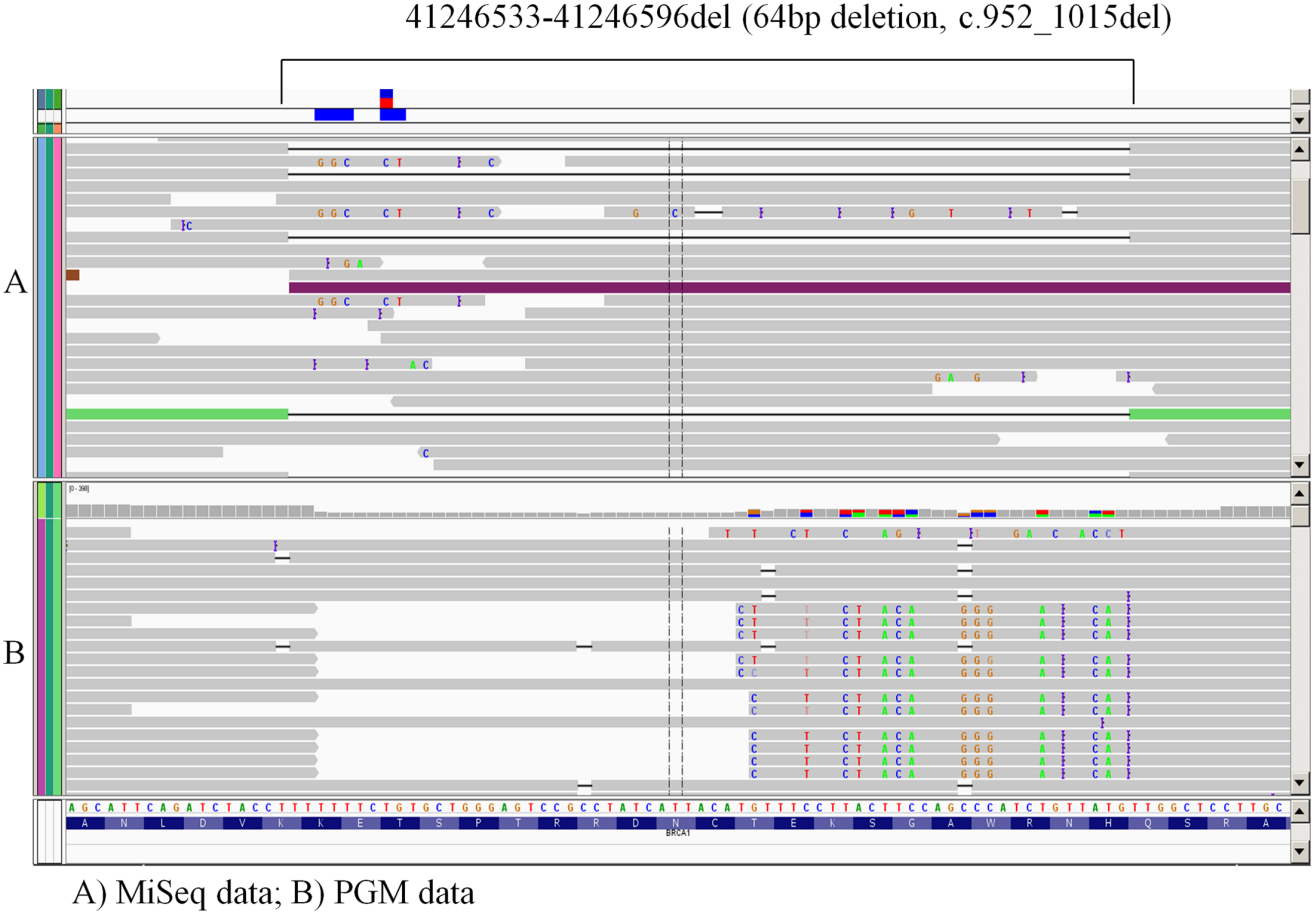
Alignment of a 64 bp-deletion (41246533-41246596del; c.952_1015del) in a validation sample. The Integrative Genomics Viewer (IGV) graphic reports show detection of the deletion using MiSeq platform with QSAP (panel A) but not the PGM platform with Torrent Suite variant calling software (panel B).

After making these adjustments to the QC metrics, the MiSeq/QSAP platform has made no further errors in more than 500 consecutive analyses. However, the PGM/Torrent Suite combination suffered 2 false-negative results for pathogenic *BRCA1* variants: a 10-base pair insertion and a 64bp deletion. Both of these pathogenic variants were detected with the MiSeq/QSAP platform. Fig 3 shows the QSAP alignment for this 64-bp deletion. Following this observation we discontinued the use of the PGM/Torrent Suite platform. To be certain that our new quality metric would ensure the identification of all variants, we began performing all MiSeq/QSAP analyses in duplicate. Duplication would ensure that any false-positives or false-negatives due to strand bias, low coverage, or library creation would be detected. In 1006 consecutive duplicate MiSeq/QSAP runs with more than 5000 variants detected, there were no discrepant results between duplicate analyses (data not shown). Therefore, we have now eliminated the duplicate run requirement.

**Fig 4:**
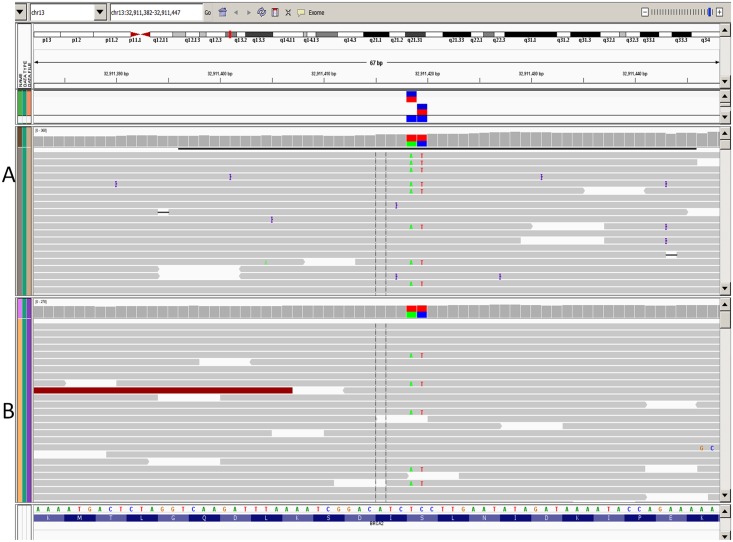
Determination of *cis* vs *trans* orientation using next-generation sequencing (NGS). The Integrative Genomics Viewer (IGV) graphic report is from a patient with 2 adjacent variants on a single DNA molecule visualized with NGS on the MiSeq/QSAP platform. The *cis* orientation is clearly visible, as each strand contains either both or neither of the mutations.

An advantage of NGS platforms over standard Sanger sequencing is their ability to determine if 2 SNPs are *cis* or *trans* in orientation. If two variants are captured in a single read (in this case less than 250 bases), they are revealed as being in *cis*. If they are captured on separate reads, then they are revealed to be in *trans*. Fig 4 shows an individual who has two point mutations in *cis*. We have already seen 2 such linked variants in the first 521 clinical samples. In addition, in routine operations, the MiSeq/QSAP platform was considerably more robust than the PGM/Torrent Suite platform. We therefore decided to discontinue the PGM/Torrent Suite platform test and are now performing duplicate MiSeq/QSAP runs for each case to determine if there are any potential problems with false-positives or false-negatives due to library formation.

There was a single discrepancy between the duplicate MiSeq runs in noted in the initial 100 duplicate samples. This was a benign polymorphism that was detected in one run, but not dectected in the second run. Examination of the sequencing data revealed that there was significant strand bias in the second sequencing run leading to 19% a variant frequency for the variant. Our QC cut-off at the time was 20%. In order to prevent reoccurrence of this error we completely revamped our QC metric for variant calling. The random fragmentation of the DNA causes each individual DNA fragment to have a unique starting and ending nucleotide. This allows the bioinformatics to uniquely identify the clones sequenced. We adjusted our QC metric to require that each base in every coding exon and 50 base pairs into the exon be sequenced from at least 20 different clones. This eliminates errors due to sequencing bias, since reads from an overrepresented clone with be ignored, and by requiring a minimum number of clones to be sequenced, heterozygotes have close to a 50% representation. After making this adjustment, no discrepancies were found in more than 1006 consecutive analyses.

### Summary

For the intial 521 clinical samples, confirmation of positive MiSeq / QSAP results was performed by the PGM / Torrent suite platform. Subsequently confirmation of results were performed by comparing duplicate MiSaq / QSAP analysis for the next 1006. We subsequently eliminated the requirement for duplicate MiSeq/ QSAP runs.

## Discussion

Our data demonstrate that neither the Illumina MiSeq sequencer with the supplied MiSeq Reporter software nor the Life Technologies PGM with the supplied Torrent Suite software are suitable for clinical laboratory sequencing of *BRCA1* and *BRCA2*. The MiSeq system’s inability to detect insertions and deletions larger than 9 bp makes it unacceptable for *BRCA* testing, as many of the described deleterious mutations are in that size range [15]. Similarly, the inability of the PGM with Torrent Suite software to detect a 10-base pair insertion and 64-bp deletion disqualifies that platform from clinical *BRCA* testing [15]. However, by combining random shearing with bait tile capture, the MiSeq platform with the bioinformatics of the QSAP alignment and allele calling software, and our quality metrics, we were able to design an assay with 100% sensitivity and specificity for *BRCA1* and *BRCA2* sequence variations in our technical validation series. Real-world performance may not reach this level of precision.

The use of NGS with bait tile exon capture offers several advantages. First, bait tile exon capture prior to NGS decreases the likelihood of false-negative results due to allele drop-out, which may occur with PCR-based methods when polymorphisms are present in amplification or sequencing primer sequences [14]. Second, with 5x redundant tiling, each exon is captured by multiple baits, further reducing the chance of a false-negative result due to individual sequence variation. A third advantage of bait tile capture versus PCR-based target enrichment methods is the avoidance of false-positive results due to amplicon bias in PCR or library formation. If a base substitution error occurs in an early PCR or library amplification cycle, the error will be propagated and result in a mixed population prior to sequencing. If an error occurs in a single amplicon, and the amplicon is preferentially sequenced, this can result in a false-positive result. With the bait tile capture approach, genomic DNA is randomly sheared to fragments of approximately 250 bp prior to bait tile capture. Library formation occurs after capture. Thus, each fragment has different 5 and 3 prime termini, and the sequence alignment software can detect if 2 reads are generated from the same fragment. Filters can be set to only accept reads from unique fragments, thereby eliminating the possibility of sequencing errors due to early PCR or library amplification errors. The selected quality control metrics require reads from at least 20 different clones, minimizing the risk of false-positive sequencing results in NGS.

Relative to Sanger sequencing, NGS also has the advantage of detecting the phase of SNPs within approximately 250 bp (i.e., the length of sheared genomic DNA fragments). Since this technology sequences a single molecule, 2 SNPs that are in *cis* orientation will appear together in the same read; if the orientation is trans, the 2 SNPs will appear in separate reads. Sanger sequencing cannot differentiate between *cis* and *trans* orientation without resorting to family studies.

In conclusion, we describe the development and validation of a rapid, high-throughput sequencing assay for the detection of *BRCA1* and *BRCA2* variants suitable for the clinical laboratory. Results from the initial 1006 clinical samples tested in duplicate with the MiSeq/QSAP combination showed no discrepant variant calls.

All sequencing data have been uploaded to the NCBI BioSample Database (www.biospecimens.samples.gov, permanent accession number is SAMN03946419).
